# Determination of causes of adult deaths using minimally invasive tissue sampling in Gandaki province of Nepal: a multicenter hospital-based study

**DOI:** 10.1186/s40001-023-01392-0

**Published:** 2023-10-07

**Authors:** Nuwadatta Subedi, Suraj Bhattarai, Sunita Ranabhat, Binita Koirala Sharma, Madan Prasad Baral

**Affiliations:** 1 Department of Forensic Medicine, Gandaki Medical College Teaching Hospital and Research Center, Gandaki Pokhara, Nepal; 2 DECODE MAUN Research Project, Gandaki Medical College Teaching Hospital and Research Center, Pokhara, Nepal; 3 Global Health Research & Medical Interventions for Development (GLOHMED), Kathmandu, Nepal; 4 Department of Pathology, Gandaki Medical College Teaching Hospital and Research Center, Pokhara, Nepal; 5 Department of Microbiology, Tribhuvan University Prithvi Narayan Campus, Pokhara, Nepal; 6 Department of Forensic Medicine, Pokhara Academy of Health Sciences, Western Regional Hospital, Pokhara, Nepal

**Keywords:** Cause of death, Complete diagnostic autopsy, Low- and middle-income countries, Minimally invasive tissue sampling, Mortality surveillance, Nepal

## Abstract

**Background:**

Minimally Invasive Tissue Sampling (MITS) has been successfully used to establish the cause of death in low- and middle-income countries, mostly in stillbirths and neonates. The objective of this study was to determine the causes of death among adults using MITS in the Gandaki province of Nepal and to find out the contribution of MITS to identify the causes of death.

**Methods:**

A multicentric hospital-based pilot study was conducted to enroll 100 cases of adult deaths. The specimens of cerebrospinal fluid, blood, brain, lungs, and liver tissue were collected utilizing MITS. These specimens underwent standard histopathological, serological, and microbiological analyses. The findings from MITS, and if available, clinical records and forensic autopsy findings were compiled and the cause of death panel identified the causes of death. The final cause of death allocated to each case was based on the WHO International Medical Certificate of Death.

**Results:**

Among a total of 100 cases enrolled during the study period, infectious cause attributed to the immediate cause of death in 77 (77%), cardiovascular in 10 (10%), neurological in 8 (8%), malignancy in two (2%), and gastrointestinal and hepatobiliary cause in one (1%) case. The mean age of the cases was 50.8 ± 15.9 years and 76 (76%) were males. MITS established the cause of death in the causal chain of events in 81(81%) cases and identified the cause of death significantly more with infectious than non-infectious causes (*p* < 0.001).

**Conclusions:**

MITS was useful in establishing the cause of death in the majority of adult deaths and the most common cause was infectious disease. Our findings suggest that MITS can be a valuable and alternative tool for mortality surveillance in low-resource settings, where complete diagnostic autopsies are less accepted or less prioritized.

## Introduction

Autopsies continue to play a significant role in improving the accuracy of mortality statistics [1]. Clinical diagnoses have shown a high rate (10–30%) of discordance with the results of complete diagnostic autopsies (CDA), which are regarded as the “gold standard” to determine the cause of death (CoD) [2–4]. However, the validity of CDA findings relies on the availability of properly equipped facilities, skilled human resources, and the quality of laboratory tests performed. The CoD cannot be determined with accuracy if adequate ancillary investigations are not performed as part of an autopsy.

In high-income countries, although on the decline, clinical autopsies are more readily performed for determining CoD [5]. However, in Nepal and many other low–middle-income countries (LMICs), an autopsy is performed for forensic cases only. Still, the cause of death remains undetermined in a high proportion (21%) of cases [6]. The data of deaths with infectious etiology are also not clearly available. Evidence has shown that there are several errors in reporting the CoD in death certificates [7], and it does not reflect adequate analysis of clinical and laboratory data or antemortem events leading to death. Therefore, autopsy and further laboratory examinations of body samples can be useful to improve the ascertainment of CoD [8].

Considering the fact that relatives of the deceased are usually reluctant to allow diagnostic CDA for the anticipated delay in the funeral process and religious belief, the use of minimally invasive tissue sampling (MITS) can overcome those constraints [9, 10]. MITS involves the sampling of different organs by the use of biopsy needles and the collection of body fluids and tissue materials for histological and microbiological analyses. Studies have shown a high concordance between MITS findings and the results of CDA [11–15]. Recently, MITS has been widely used in the LMICs as a reliable tool to determine the CoD both in community and hospital settings [16, 17]. The procedure has also been safely conducted in COVID-19 cases [18]. As it is a less time-consuming procedure and there are no incisions that would disfigure the body, relatives of the deceased seem to culturally accept this technique too [19].

Collecting accurate data on mortality and the causes of death can be useful for evidence-based planning and implementation of public health policies and programs in resource-limited settings. We used MITS in an attempt to better identify the causes of death in the study population.

## Materials and methods

The objective of this study is to determine the causes of deaths in the study population and to find out the contribution of MITS to identify the causes of death.

A multicentric hospital-based cross-sectional study was conducted at three health facilities of Gandaki Province of Nepal: Gandaki Medical College (GMC), Pokhara Academy of Health Sciences (PAHS), and Damauli Hospital, Tanahun from November 2019 to January 2021, with a brief pause from 20th March to 6th July 2020 due to COVID-19 lockdown. The study cases were adult deaths (> 18 years age), either clinical cases (who had died in the emergency department or in-patient treatment or deceased prior to hospital arrival but without having indication for forensic autopsy) or forensic cases (deceased cases for the purposes of forensic autopsies as per the prevailing laws of the nation). In forensic cases, an autopsy is conducted following the national protocol and is legally mandatory and we had performed MITS before the cases were subjected to autopsy. In Nepal, natural deaths are also subjected to forensic autopsy if there is any doubt of foul play, in unidentified bodies and if CoD is not determined clinically and is needed mainly for insurance purposes. The forensic autopsies are mainly limited to gross examination and histopathological and microbiological investigations are not routinely performed. This was designed as a pilot study with a target number of 100 cases to utilize the MITS and observe its contribution in determining the CoD among the adult deceased in our settings. The case enrollment flowchart opted by the research project is presented elsewhere [20].

At each study site, the hospital staff were informed of the research project, and they would notify the study team whenever a potential case was identified. The study team offered grief counseling to the nearest relatives of the deceased and informed them about the MITS procedure. Written informed consent was obtained from the nearest relatives before enrolling in each case. The cases included adult deaths that occurred during the study duration for which consent was obtained. Deaths directly attributed to trauma were excluded as well as cases that had evidence of decomposition and autolysis.

Data were collected from the nearest relatives using a structured enrollment questionnaire which included demographics, duration of illness, treatment history for the current and past illness, and underlying chronic illnesses if any. The study team also reviewed clinical notes of the cases which were treated in the hospital. The vital signs, investigation reports, clinical diagnosis, laboratory tests, medications or procedures performed, progress during the hospital stay, and death certificate (if issued) were noted. All findings were entered into the standard case report form. Post-mortem laboratory findings were also entered in the structured forms prepared for the study. For each case, a unique identification code (study ID) was used for sample collection, processing, laboratory tests, result reporting, and determination of the cause of death. Two forensic pathologists involved in sample collection had received 6 days of hands-on training on standard sample collection procedures of MITS at a specified training center designated by the MITS Surveillance Alliance.

### Postmortem collection of specimens using MITS

The MITS procedure was conducted by a trained forensic pathologist with the assistance of a technician. The procedure commenced by evaluating external parts of the body for the presence of any lesions, growths, and abnormalities. The natural orifices were checked for any discharge. All the lesions, if any, were identified and noted. The bodies were initially washed with clean tap water. The areas of the body to be punctured for MITS were disinfected with 96% alcohol and iodine solution, allowing 5 min for each to act on the surface of the body for adequate disinfection for the microbiological analyses and the skin was allowed to air dry. The collection of samples was done following the standard procedure for MITS [21]. We collected 5 to 10 mL of cerebrospinal fluid (CSF) using18G spinal puncture needle by occipital approach, 15 to 20 mL of blood by 18G intramuscular needle and 20 mL syringe using the supraclavicular approach, brain tissue samples for histology, lung tissue samples for microbiology and histology and liver tissue sample for histology using MITS.

### Brain tissue samples for histology

Bard Monopty 16G 160 mm was used for occipital puncture to collect brain samples. Six samples of brain tissues were collected and transferred to a formalin jar. To reach the brain parenchyma from the anterior approach, the cribriform plate was perforated using a bone-marrow trephine kit. Then, using the same needle used to collect the brain samples from occipital puncture, six brain tissue samples from different depths and directions were collected from the trans nasal approach inserting through the perforation in the cribriform plate.

### Lung tissue samples for microbiology

The puncture was made with the Bard Monopty 16G 100 mm needle in the mid-axillary line on the right and left separately to different directions and depths to retrieve 12 samples each. The first six samples were transferred to Brain Heart Infusion (BHI) media in an aseptic manner and the next six samples from each lung were transferred to a sterile tube with normal saline to send for GeneXpert analysis for *Mycobacterium tuberculosis*.

### Lung tissue samples for histology

The same needle which was used for Lung/Thorax Microbiology was used in a similar manner as for microbiology to retrieve six samples from each lung from different depths and directions. The samples were transferred to the prelabeled formalin jars for each side.

### Liver tissue samples for histology

To obtain histopathological samples from the liver, we performed a puncture with the Bard Monopty 16G 100 mm needle in the mid-axillary line, in one of the three last intercostal spaces. The needle was oriented 30º in a cranial and 15º in a posterior direction and should penetrate 2–4 cm depending on the size of the body. We obtained six liver biopsies using the same entry point and changing the direction and depth. The samples were transferred to a prelabeled liver formalin jar.

### Additional specimens in selected cases

Heart tissue samples for histology were collected from cases with cardiac history by obtaining either from clinical records or from relatives. It was done using the Bard Monopty 16G 100 mm needle by puncture through the left thoracic region at the 5th intercostal space in a parasternal point. In addition, when there was any skin lesion, the sample was taken by punch biopsy and transferred to an extra formalin jar. After the procedure was completed, any areas for excessive seepage or bleeding were checked and the appropriate packs were placed. The bodies were washed and wrapped in a sheet of cloth to hand over to the relatives.

After MITS was completed, the forensic cases underwent autopsy, following the routine national protocol. The information obtained from the forensic autopsy reports was also collected to utilize in the determination of the cause of death (DeCoDe) process.

### Laboratory procedures

The CSF samples were examined microscopically by Gram stain. They were further cultured on blood agar and chocolate agar plates and incubated at 35 °C for 7 days. The 10 mL blood placed into EDTA tubes was centrifuged to separate the plasma supernatant. Plasma samples were tested for the presence of antibodies against human immunodeficiency virus (HIV) 1/2, hepatitis B virus (HBV) surface antigens, and antibodies against hepatitis C virus (HCV) using rapid diagnostic kits. Tests for Dengue (NS1, IgM, IgG), Leptospira (IgM, IgG), Brucella abortus Antigen, Malaria (Pf, Pan Malaria) were also done. The lung tissue samples were subjected to GeneXpert to detect the presence of *Mycobacterium tuberculosis*. The details of microbiological procedures used in this research are presented elsewhere [20].

The tissue specimens collected for histological analysis were fixed with 10% neutral buffered formalin for 4 h and then passed into a tissue processor for 8 h, embedded in paraffin, and cut into four-micron sections, which were then stained with hematoxylin and eosin (H&E) as per standard procedures. The H&E sections of all tissues were initially evaluated, and whenever necessary, ancillary histochemical tests, i.e., Ziehl–Neelsen and Periodic Acid Schiff were also performed. All the cylinders of tissues collected in each block were carefully evaluated, recording all the organs present under a microscope Olympus CX 23, in 4×, 10×, 40× and 100× magnification.

### Determination of the cause of death

**Fig. 1:**
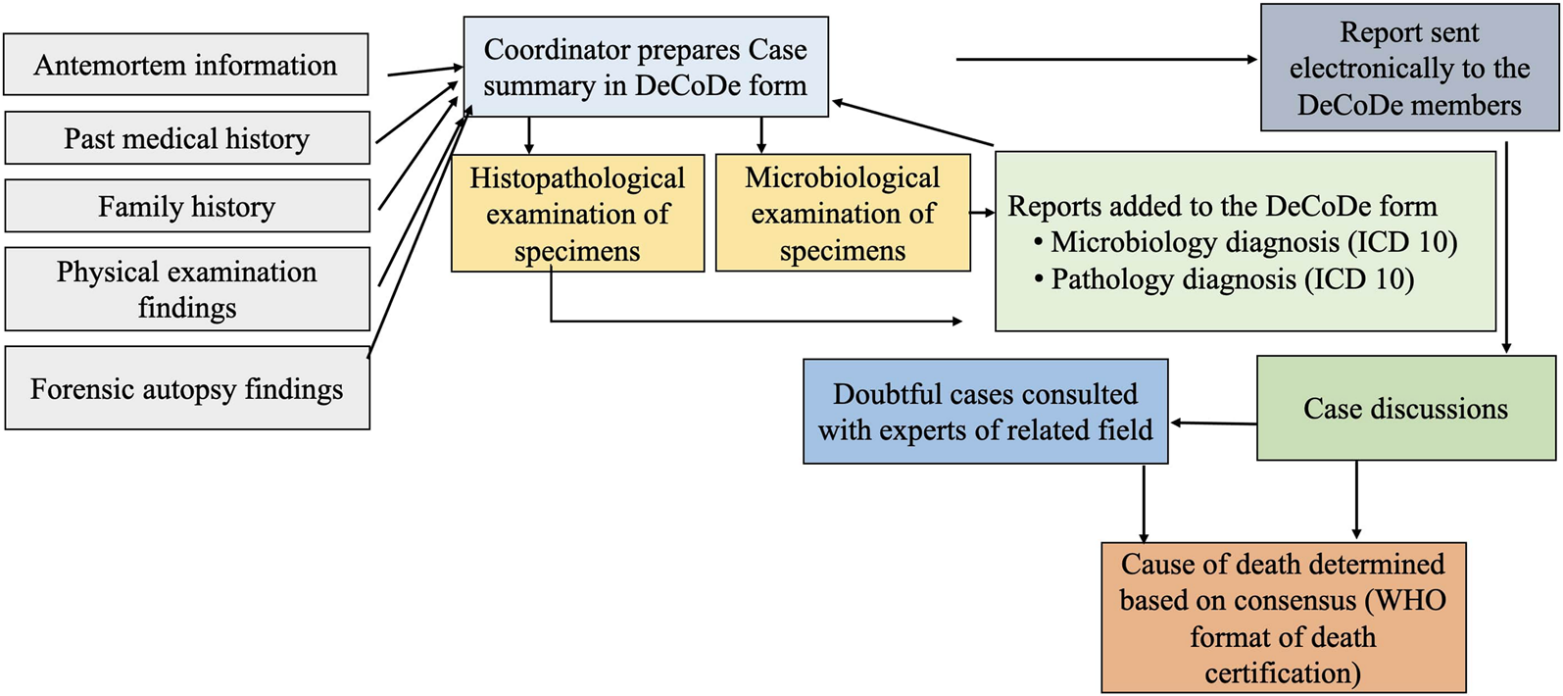
Schematic diagram of the procedure of determination of cause of death. DeCoDe: determination of cause of death, ICD: International Classification of Disease

We formed a panel of experts called the DeCoDe panel which consisted of five core investigators (two forensic pathologists, one microbiologist, one clinical pathologist, and one social science/tropical and infectious disease specialist) and a consultant physician. The coordinator of the project (NS) prepared a summary of case details using a standard format including antemortem information of the deceased, past medical history (including information of morbidity and mortality in family members), physical examination findings, autopsy findings in forensic cases, and sample collection details and sent to the panel electronically. The histopathologist and the microbiologist were blinded on the test findings to each other until the case discussion began during the scheduled DeCoDe meetings and other asynchronous online meetings. This was done to eliminate bias in diagnosis. All the case diagnoses were coded according to the International Classification of Disease 10^th^ edition (ICD 10) protocol [22]. The causes of death allocated to each case were based on the WHO International Medical Certificate of Death [23] and were determined based on the consensus among panel members. For any ambiguity or if in-depth clinical consultation was needed during the discussion in the panel, additional subject experts were invited to the meetings to discuss the specific case(s) and finalize the CoD. In cases where more than one condition led to death, the entire causal chain leading to death was documented according to the format of the standard death certificate which includes immediate cause, antecedent causes (events in between immediate and underlying cause), and the underlying cause. Other causes which contributed to death but were not directly responsible for the causal chain of events were identified as contributing causes. The DeCoDe process followed by the research project is shown in Fig. 1.

Once determined by the DeCoDe panel, the causes of death were categorized as infectious, malignancy, others including the cardiovascular, respiratory, central nervous system, and non-conclusive. For this categorization, the CoD mentioned in the immediate cause of the causal chain was considered. We collected information from all the available sources for determination of CoD in addition to data from MITS. When the causes of death identified in the death certificate for the case were based on analysis of the samples obtained from MITS with or without correlation of antemortem information, it was considered as “contributed by MITS”, whereas if the causes of death were just based on the antemortem information, clinical data, or from forensic autopsy findings, without any contribution from the analysis of MITS samples, they were considered “not contributed by MITS”.

### Ethical approval

Ethical approval was obtained from the Ethical Review Board of Nepal Health Research Council (Reg no. 511/2019, approval date: August 22, 2019) prior to enrolling cases. Administrative approvals were also taken from respective institutions; Gandaki Medical College, Pokhara Academy of Health Sciences, and Damauli Hospital.

### Data entry and analysis

All data were entered into Microsoft Excel and statistical analyses were done using Statistical Package for Social Sciences (SPSS version 16.0). Frequency, percentage, mean, and standard deviation were used to present the data. Fisher’s exact test was used to analyze the differences in the capacity of MITS to establish the cause of death in infectious and non-infectious causes. *p* value of < 0.05 was regarded as significant.

### The role of the study sponsor

The study was supported by the MITS Surveillance Alliance with funding from the Bill and Melinda Gates Foundation which did not have direct involvement in designing the study, although feedback regarding the study protocol, laboratory investigations, and procedures, and the DeCoDe protocol was provided. The Alliance also provided a 5-day in-person training to members of the research team in an established training hub. Furthermore, an implementation visit was conducted by a team from the Alliance after we enrolled the first four cases to observe the progress, guide the team in the collection and analysis of samples, upload histology slides to the Quality Assurance (QA) portal and provide feedback. The Alliance also conducted regular online technical working group meetings to enhance the capacity of the members. The QA of the MITS procedure was done by evaluating the histology slides uploaded in a predefined systematic manner to the QA portal on the website of the Alliance.

## Results

A total of 100 cases were enrolled in the study period. Clinical cases: a total of 257 deaths were reported from the study sites in the study duration, of which 200 met the inclusion criteria; 130 of them were not approached for consent as the case enrollment was affected by the COVID-19 pandemic, and priority was given to patient care. In addition, the uncertainties of safety issues in the initial days of the pandemic and unavailability of adequate safety measures affected the case enrollment. Only 70 were approached for enrollment of which 22 consented and were enrolled, leading to a consent rate of 31·5%. All the eligible clinical cases could not be approached for consent because of the interruption of research activities caused by the COVID-19 pandemic. Forensic cases: a total of 501 forensic autopsies were conducted at the study sites during the study period among which 423 forensic cases were known traumatic or violent deaths so were excluded, whereas 78 met the inclusion criteria; all of them consented and were enrolled into the study. Among the 100 cases, 76 (76%) were males and 24 (24%) females. The mean age of the cases was 50·8 ± 15·9 years. The demographic characteristics of the cases are represented in Table 1.

**Table 1:** Demographic characteristics of study cases

Variables	Sex		Total (*N* = 100) Frequency (%)
	Males (*n* = 76) Frequency (%)	Females (*n* = 24) Frequency (%)	
*Age in years*			
18–45	34 (44.7)	10 (41.7)	44 (44)
46–65	30 (39.5)	9 (37.5)	39 (39)
> 65	12 (15.8)	5 (20.8)	17 (17)
*Marital status*			
Married	47 (61.8)	15 (62.5)	62 (62)
Unmarried	11 (14.5)	2 (8.3)	13 (13)
Widowed/divorced	9 (11.4)	7 (29.2)	16 (16)
Not known	9 (11.4)	0 (0)	9 (9)
*Occupation*			
Home maker	11 (14.5)	18 (75.0)	29 (29)
Manual worker	21 (27.6)	1 (4.2)	28 (28)
Unemployed	20 (26.3)	3 (12·5)	23 (23)
Self employed	6 (7.9)	1 (4·2)	7 (7)
Office work	6 (7.9)	0 (0)	6 (6)
Student	1 (1.3)	1 (4.2)	2 (2)
Not known	5 (6.6)	0 (0)	5 (5)
*Ethnicity*			
Indo-Nepalese	58 (76.3)	17 (70.8)	75 (75)
Tibeto-Nepalese	18 (23.7)	7 (29.2)	25 (25)
Smoker	45 (59.2)	17 (70.8)	62 (62)
Smokeless tobacco user	39 (51.3)	7 (29.2)	46 (46)
Alcohol user	59 (77.6)	14 (58.3)	73 (73)
Other drugs of abuse	4 (5.3)	0 (0)	4 (4)
*Place of death*			
Home/road	57 (75)	19 (79.2)	76 (76)
Hospital facility	19 (25)	5 (20.8)	24 (24)

**Fig. 2:**
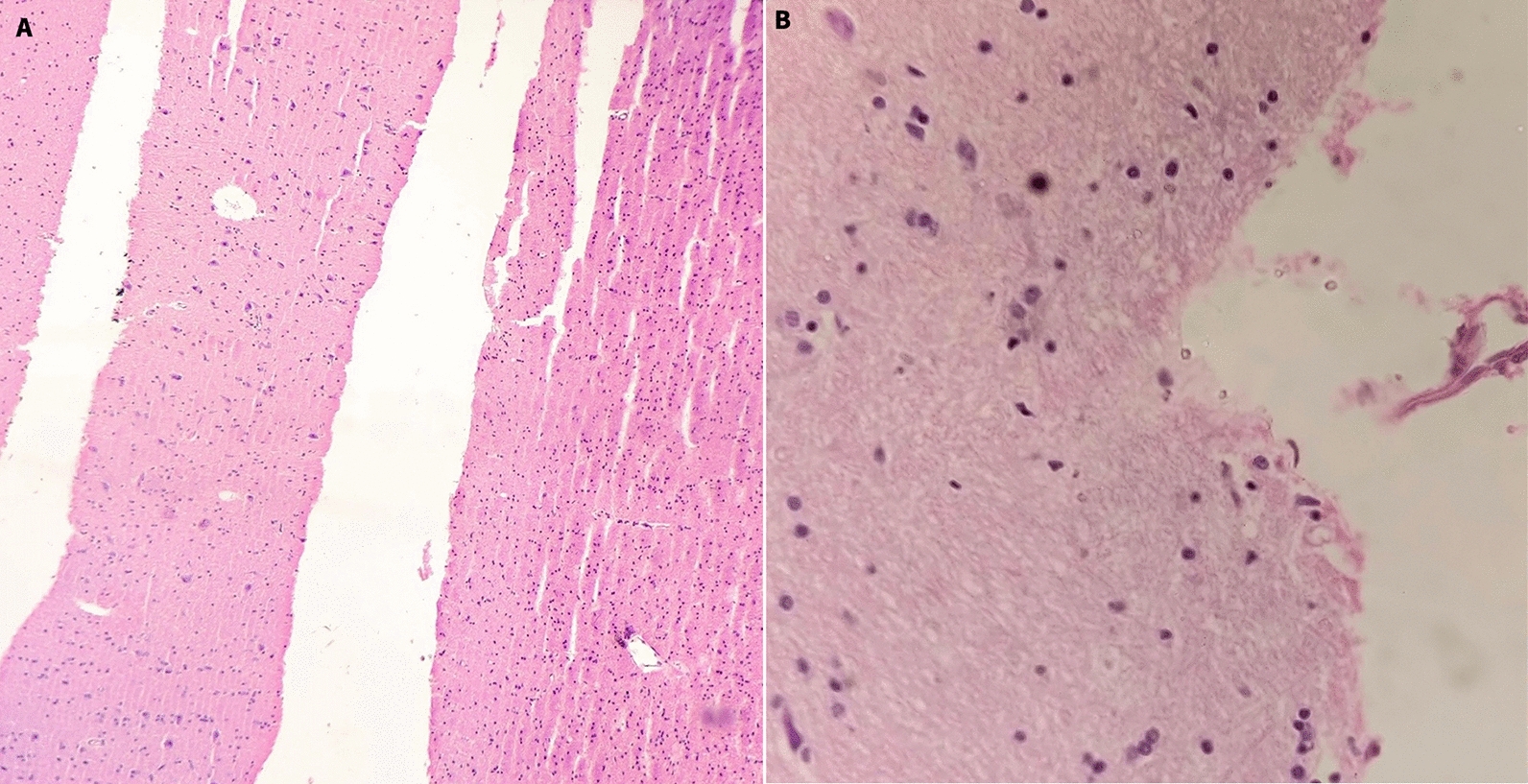
Histology of brain tissues retrieved by MITS. **A** Normal brain tissue (H&E ×100). **B** Section showing cryptococci (H&E ×400)

**Fig. 3:**
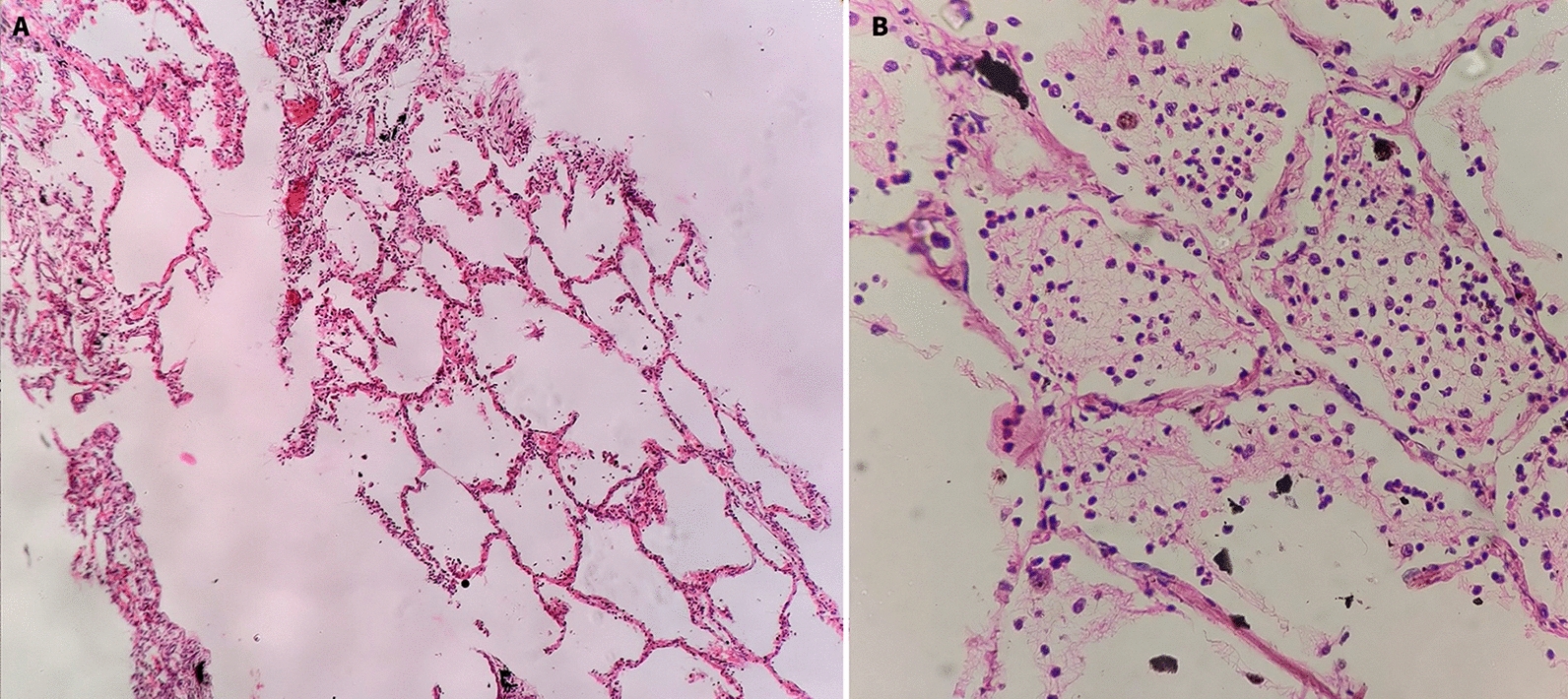
Histology of lungs tissues. **A** Section with normal alveolar spaces (H&E ×100). **B** Alveolar spaces filled with mixed inflammatory cells and fibrin suggestive of pneumonia (H&E ×400)

**Fig. 4:**
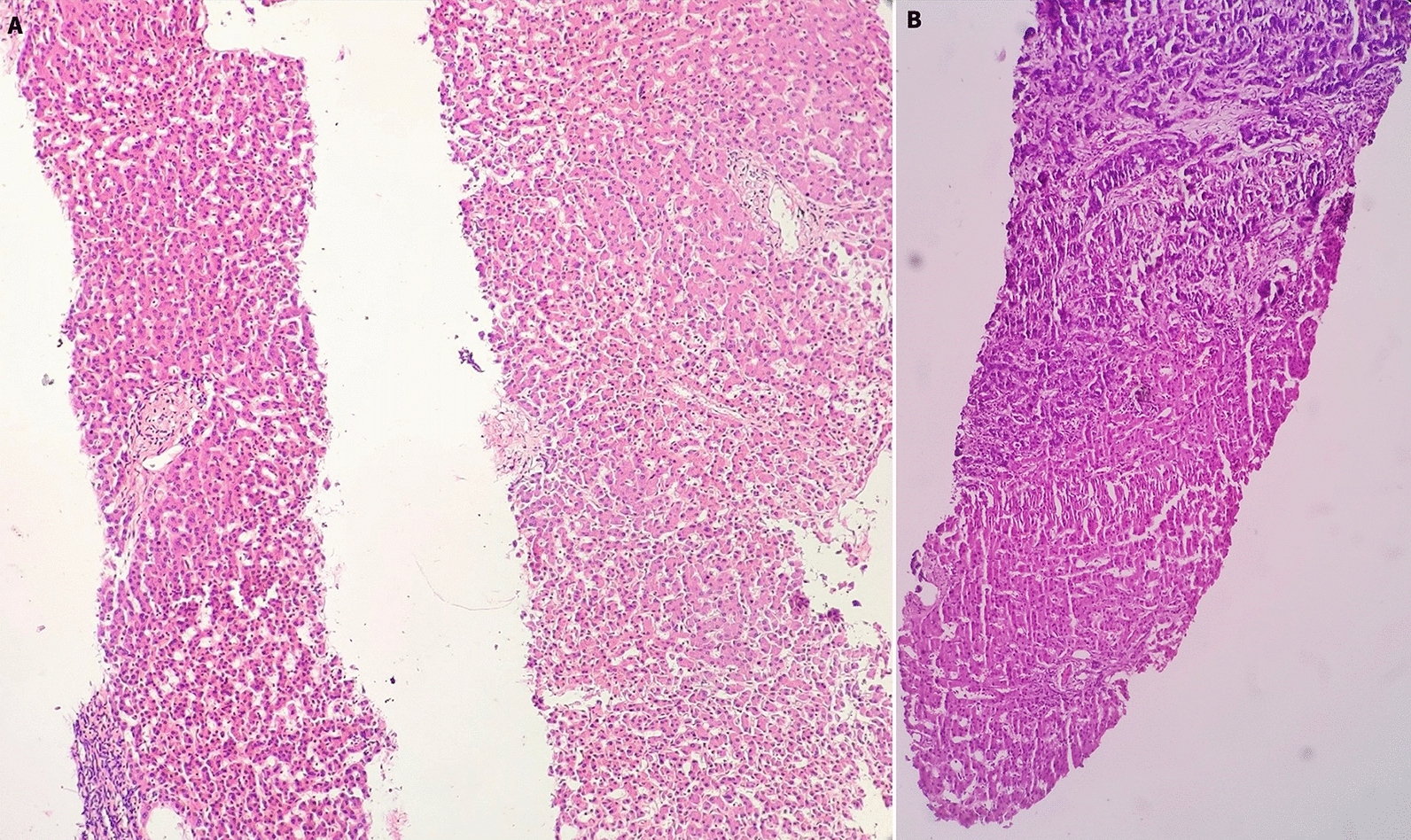
Histology of liver tissues. **A** Section with two cores of liver tissues of unremarkable histology (H&E ×100). **B** Tissue with tumor in the upper part of the section and lower normal portion (H&E ×100)

The histopathology investigations diagnosed cryptococcosis from the brain tissues in one (1%) case, otherwise, the brain was unremarkable in the remaining 99 (99%) cases. Among pathologies in lung tissues, anthracosis was evident in 81 (81%) cases, followed by pneumonia in 33 (33%), tuberculosis in three (3%), and malignancy in one (1%) case. Steatosis was diagnosed from the liver tissues in 33 (33%) and steatohepatitis in five (5%) cases. Liver cirrhosis was diagnosed in four (4%) and hepatocellular carcinoma in two (2%) cases. Some of the histopathological diagnoses made from the specimens obtained by MITS, and the corresponding normal findings are shown in Figs. 2, 3 and 4. Serological tests revealed brucellosis in six (6%), dengue in four (4%), and HIV and Hepatitis B in two (2%) cases each.

The etiological microorganisms responsible for the infectious cause of death and their antimicrobial resistance pattern are presented in detail elsewhere [20]. Out of 77 (77%) cases that were identified to have bacterial infections as the cause of death, *Klebsiella oxytoca* was identified as the commonest etiological organism (in 36 cases; causing pneumonia in 23, sepsis in 12, and meningitis in one case), followed by *Klebsiella pneumoniae* (in 22 cases contributing to pneumonia in 15 and sepsis in seven cases). Other common etiological agents leading to mortality were *Staphylococcus aureus* in 11, *Proteus vulgaris* in 10, *Acinetobacter species* in nine, *Proteus mirabilis,* and *Citrobacter freundii* each in eight, and *Escherichia coli* in seven cases.

**Fig. 5:**
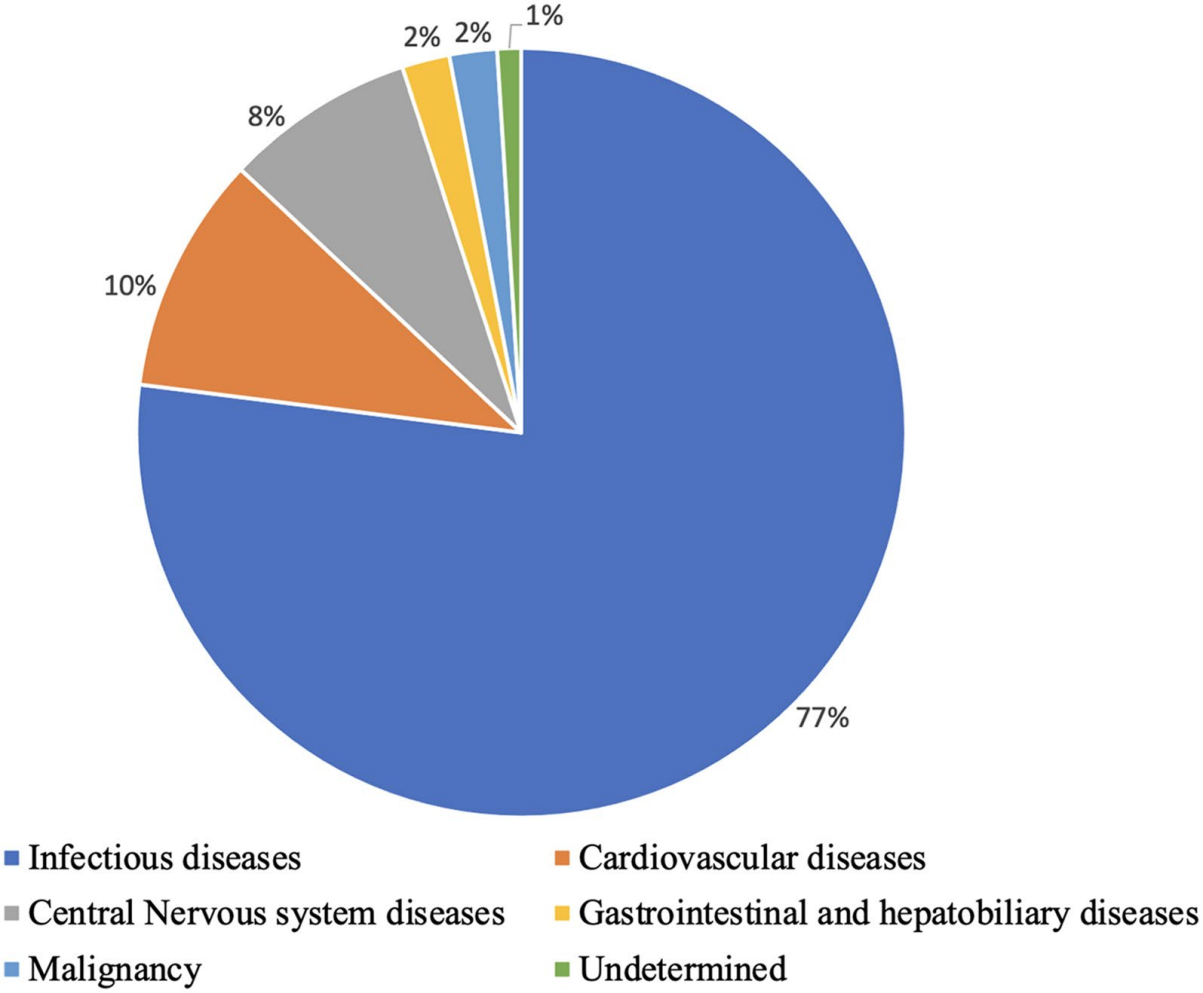
Distribution of category of immediate cause of death

All identified causes of death in the causal chain of events (Part I of the WHO International Medical Certificate of Death) considering the findings from MITS and others (clinical diagnoses and forensic autopsy findings) are presented in Table 2. Bacterial pneumonia was identified in the causal chain in 68 (68%) cases, followed by sepsis in 35 (35%) cases. The distribution of the category of immediate causes of death is shown in Fig. 5. Infectious diseases were the most common immediate cause of death in 77 (77%) cases followed by cardiovascular diseases in 10 (10%) cases.

**Table 2:** All identified causes of death in the causal chain of events in the study cases

Causes of death	Immediate cause	Antecedent cause	Underlying cause	Total (*N* = 100) Frequency (%)
*Infectious diseases*				
Bacterial pneumonia	35	31	2	68 (68)
Sepsis	33	2	0	35 (35)
Pulmonary tuberculosis	2	1	2	5 (5)
Bacterial meningitis	3	1	0	4 (4)
Brucellosis	2	0	1	3 (3)
HIV	0	1	1	2 (2)
Typhoid fever	1	0	0	1 (1)
Disseminated cryptococcosis	1	0	0	1 (1)
Hepatitis B	0	1	0	1 (1)
*Cardiovascular diseases*				
Atherosclerotic heart disease	5	1	0	6 (6)
Myocardial infarction	2	0	0	2 (2)
Hypertrophic cardiomyopathy	2	0	0	2 (2)
Alcoholic cardiomyopathy	1	0	0	1 (1)
Hypertensive heart disease	0	5	0	5 (5)
Functional disturbances following cardiac surgery	0	1	0	1 (1)
Thromboembolism	0	1	0	1 (1)
Multiple valve diseases	0	0	1	1 (1)
*Central nervous system diseases*				
Meningeal hemorrhage	6	0	2	8 (8)
Intracerebral hemorrhage	2	0	0	2 (2)
Cerebral infarction	0	0	1	1 (1)
*Gastrointestinal and hepatobiliary diseases*				
Alcoholic cirrhosis of liver	0	2	1	3 (3)
Alcoholism	1	1	0	2 (2)
Alcoholic liver disease	0	2	0	2 (2)
Esophageal varices with bleeding	1	0	0	1 (1)
Alcoholic hepatic failure	0	1	0	1 (1)
Cirrhosis of liver	0	1	0	1 (1)
Alcoholic hepatitis	0	0	1	1 (1)
*Malignancy*				
Malignant neoplasm of lung	1	0	0	1 (1)
Malignant neoplasm of liver	1	0	0	1 (1)
Malignant neoplasm of pancreas	0	1	0	1 (1)
Malignant neoplasm of uterus	0	0	1	1 (1)
*Others*				
Chronic obstructive pulmonary disease (COPD)	0	1	0	1 (1)
Acute exacerbation of COPD	0	1	0	1 (1)
Acute renal failure	0	1	0	1 (1)
Extrapyramidal and movement disorder	0	1	0	1 (1)

When we analyzed the role of MITS alone in establishing the CoD, it was evident that findings from MITS only established the immediate cause in 79 (79%) and antecedent and underlying causes in 45 (45%) (Table 3). The data generated from MITS alone established the CoD in the causal chain of events in 81 (81%) cases. In the cases where the data from MITS alone was not sufficient to determine the cause in the causal chain of events, it was established either by the information obtained from forensic autopsy or from clinical data in 18 (18%) cases, whereas the cause was left undetermined in one case. The data obtained from MITS findings were incorporated to the contributory cause (Part II of the WHO death certificate) in 89 (89%) cases. Therefore, considering the usefulness of MITS findings alone in the death certificate, it added some information to it in 95 (95%) of cases when the contributory causes were also included.

**Table 3:** Contribution of findings from MITS alone to determine cause of death

Cause of death	Contribution of MITS for the cause of death	
	Yes *n* (%)	No *n* (%)
Immediate cause	79 (79)	21 (21)
Antecedent and underlying cause	45 (45)	55 (55)
Causal chain of events (Part I of the WHO International Medical Certificate of Death)	81 (81)	19 (19)
Contributory cause	89 (89)	11 (11)
Overall	95 (95)	5 (5)

Furthermore, when we classified the mortality cases as having infectious causes in any level of the casual chain in part I of the WHO International Medical Certificate of Death (either immediate, antecedent, or underlying causes), and non-infectious or undetermined, we found that relative odds of deaths due to the infectious cause was 346.5-fold (246.5%) greater compared to deaths due to non-infectious cause when determined by MITS [95% CI 33.36, 14,587.25; *p* value < 0.001]. In other words, MITS is more suitable for ascertaining the CoD when deaths occurred due to infectious diseases (Table 4).

**Table 4:** Contribution of MITS to determination of causes of death in infectious and non-infectious causes in the causal chain of events

Cause of death	Contribution of MITS in finding cause of death		Total	Odds ratio	95% confidence interval	*p* value
	Yes	No				
Infectious cause of death	77	1	78	346.5	33.36–14,587.25	< 0.001
Non-infectious cause of death or undetermined	4	18	22			
Total	81 (81%)	19 (19%)	100			

## Discussion

This pilot study was conducted with objectives of determining the causes of deaths in the study population and to find out the contribution of MITS to identify the causes of death. The CoD is not satisfactorily established in low-resource settings. In Nepal, deaths which are not subjected to forensic autopsy as per the existing national rules or deaths, which occur outside of healthcare facilities, are never examined. Clinical autopsies are not usually performed either. Even if autopsies are performed, accurate diagnosis is not always made [6], due to several constraints, such as unavailability of facilities to conduct clinical autopsies, lack of trained pathologists, and less priority by clinicians and relatives of the deceased to establish the cause of death. As a result, it is possible that the largely underestimated burden of diseases in the community has hampered/reduced the effectiveness of disease prevention strategies.

In the setting ,where intervention research is rarely done, the use and acceptability of MITS were initially challenged by the research stakeholders–hospital management, clinicians, law enforcement, family or relatives of deceased, community leaders. To overcome this, the study team organized awareness programs targeted to these stakeholders. Gradually, the technique was accepted, and with the use of well-established consenting procedures and research strategies [24], our targeted number of cases could be enrolled. The evidence has shown that the relatives of the deceased who would have otherwise rejected CDA, tend to accept MITS [25]. Nearly, one-third of the approached family members or relatives of the deceased in clinical cases consented for MITS, which implies the feasibility of the procedure in autopsy hesitant settings. The experience and lessons learned during the implementation of MITS in Nepal along with the similarities and differences with other similar settings are presented elsewhere [26].

In line with the objectives of the study, we found that the implementation of MITS has been helpful in establishing the CoD among the adult population. This is because the findings from MITS alone contributed to establishing the cause of death in the causal chain of events (part I of the WHO death certification) in 81% of cases. In an observation from Mozambique, the CoD was identified through MITS involved histological and/or the microbiological analysis in 83% cases,_9_ which is approximate to our findings; CoD determination was 89.2% in another study from Mozambique among the adult population [11]. Implementation of MITS along with molecular diagnostics was useful in identifying at least one CoD in 98% cases among under-5 mortality and stillbirths, as reported by studies conducted at five sites in Mozambique, South Africa, Kenya, Mali, and Bangladesh [27]. Based on this evidence, researchers made suggestions for the utilization of MITS globally and in all age groups [28]. In addition to the findings from MITS, the use of clinical data and findings from the forensic autopsy were useful establishing the causes of deaths in 99 (99%) cases in our observation. Clinical data have been proved to be highly informative in mortality surveillance if recorded properly [12]. The forensic autopsies are not routinely backed up by laboratory investigation in our set up and many causes are missed, mainly in deaths due to diseases. The laboratory investigations performed as a part of this study were successful in identifying the existing diseases and helpful to establish the COD in the study cases.

Most of the deaths were attributed to infectious causes in our study, which is similar to the observation from Mozambique [11]. It is established by studies conducted elsewhere too, that MITS can detect infectious causes better than non-infectious causes [10]. Since this study enrolled more community deaths than hospital deaths (76% vs. 24%), mortality due to non-communicable diseases or conditions in the hospital setting could have been missed [20]. Moreover, it is always beneficial to correlate autopsy results with clinical information and other ancillary findings. The CNS causes identified in our study cases were either from the clinical data or during the forensic autopsy in natural deaths and none were from the MITS findings. In one case, tissue sample from the lungs during MITS was useful to diagnose hypertrophic cardiomyopathy as heart tissue was incidentally retrieved, while all the other cardiovascular conditions identified as the CoD in our study were obtained from clinical data or during the forensic autopsy.

In addition to the findings related to the objectives of the study, other relevant findings were also noted. The study was helpful in establishing some conditions which were never diagnosed during the lifetime of the deceased. Four cases of tuberculosis, two cases of HIV and Hepatitis B each, and one case of malignancy were never diagnosed earlier. Sharing more accurate information on the CoD can be important to the family members of the deceased, particularly for significant infectious diseases. In a case of disseminated cryptococcosis with HIV diagnosed postmortem by MITS, one of the family members was tested HIV positive and was advised to seek medical counseling and treatment according to the national protocol [29]. The family members of TB infected cases were also provided tele-counseling on the need to consult their physician for follow-up and treatment.

The study has generated valuable evidence on mortality profile among the adult population in the Gandaki Province of Nepal, setting up a foundation for larger studies to determine the local burden of diseases. In the context, where CDA is seldom done to investigate the cause of natural or clinical deaths, MITS can be a reliable and cost-effective alternative method, particularly in clinical cases with probable infectious cause [20, 26, 30]. As per the WHO estimates, only around half of the deaths are attributed to accurate cause during death registration and most of the underestimation of disease burden occurs in Africa and Asia [31]. The use of MITS can overcome such deficiencies by generating vital evidence that informs disease prevention policies.

The strengths of this study include the successful incorporation of MITS in province-level mortality surveillance, for the first time in Nepal, and enrollment of clinical death cases from hospitals, where CDA is seldom performed. The study also utilized the findings of extensive laboratory tests, to investigate the accurate CoD, which are not routinely done in the local context. There are a few limitations of this study too. The sample size was limited, as the study was designed to meet the target number of a pilot study. There was a lack of detailed antemortem clinical records, mostly in deaths that occurred outside the hospital. Moreover, case enrollment could not be done from March to July 2020 because of the nationwide lockdown imposed by the government amid the COVID-19 pandemic.

## Conclusion

MITS was successful in establishing the cause of death and was more effective in detecting infectious causes. Most of the mortality cases among the adult population were identified to have an infectious cause with bacterial pneumonia and sepsis as the most common diseases leading to death. MITS can be a valuable tool in determining the cause of death in low-resource settings, where CDA is not possible due to several constraints.

## Data Availability

The data sets used and/or analyzed during the current study are available from the corresponding author on reasonable request.

## Abbreviations

BHI: Brain Heart Infusion
CDA: Complete diagnostic autopsies
COPD: Chronic obstructive pulmonary disease
CoD: Cause of death
COVID-19: Coronavirus Disease 2019
CSF: Cerebrospinal fluid
DeCoDe: Determination of cause of death
EDTA: Ethylenediamine tetra-acetic acid
GMC: Gandaki Medical College
HBV: Hepatitis B virus
HIV: Human immunodeficiency virus
H&E: Hematoxylin and eosin
ICD: International Classification of Disease
LMICs: Low–middle-income countries
MITS: Minimally Invasive Tissue Sampling
PAHS: Pokhara Academy of Health Sciences
QA: Quality Assurance
SPSS: Statistical Package for Social Sciences
TB: Tuberculosis
WHO: World Health Organisation
